# Hepatic Oleate Regulates Insulin-like Growth Factor-Binding Protein 1 Partially through the mTORC1-FGF21 Axis during High-Carbohydrate Feeding

**DOI:** 10.3390/ijms232314671

**Published:** 2022-11-24

**Authors:** Lucas M. O’Neill, Yar Xin Phang, Zhaojin Liu, Sarah A. Lewis, Ahmed Aljohani, Ayren McGahee, Gina Wade, Mugagga Kalyesubula, Judith Simcox, James M. Ntambi

**Affiliations:** 1 Department of Biochemistry, University of Wisconsin-Madison, 433 Babcock Drive, Madison, WI 53706, USA; 2 College of Science and Health Professions, King Saud Bin Abdulaziz University for Health Sciences, Riyadh 11564, Saudi Arabia; 3 King Abdullah International Medical Research Center (KAIMRC), Riyadh 11564, Saudi Arabia; 4 Department of Nutritional Sciences, University of Wisconsin-Madison, 1415 Linden Drive, Madison, WI 53706, USA

**Keywords:** stearoyl-CoA desaturase-1, insulin-like growth factor-binding protein 1, oleic acid, stearic acid, fibroblast growth factor 21, mechanistic target of rapamycin

## Abstract

Stearoyl-CoA desaturase-1 (SCD1) catalyzes the rate-liming step of monounsaturated fatty acid biosynthesis and is a key regulator of systemic glucose metabolism. Mice harboring either a global (GKO) or liver-specific deletion (LKO) of *Scd1* display enhanced insulin signaling and whole-body glucose uptake. Additionally, GKO and LKO mice are protected from high-carbohydrate diet-induced obesity. Given that high-carbohydrate diets can lead to chronic metabolic diseases such as obesity, diabetes, and hepatic steatosis, it is critical to understand how *Scd1* deficiency confers metabolically beneficial phenotypes. Here we show that insulin-like growth factor-binding protein 1 (IGFBP1), a hepatokine that has been reported to enhance insulin signaling, is significantly elevated in the liver and plasma of GKO and LKO mice fed a low-fat high-carbohydrate diet. We also observed that the expression of hepatic *Igfbp1* is regulated by oleic acid (18:1n9), a product of SCD1, through the mTORC1-FGF21 axis both in vivo and in vitro.

## 1. Introduction

Obesity is prevalent in society and is a known predisposition for several diseases, including type 2 diabetes, cardiovascular disease, hypertension, and cancer [1]. Obesity is influenced by a combination of genetic, dietary, and environmental factors [1,2]. Currently, it is estimated that more than 70% of adults are overweight or obese in the United States of America (U.S.) and by 2030, more than half of the U.S. population will have obesity [3,4]. Consumption of excess carbohydrates increases de novo lipogenesis and is one aspect that can lead to obesity [5]. One factor that regulates weight gain in response to excess dietary carbohydrates is stearoyl-CoA desaturase-1 (SCD1). Remarkably, targeted disruption and pharmacological inhibition of SCD1 have been shown to prevent obesity in mice consuming a low-fat high-carbohydrate diet (HCD), but the mechanism of protection has not been fully elucidated [6,7].

SCD1 is an endoplasmic reticulum (ER) trans-membrane enzyme that catalyzes the rate-limiting step of monounsaturated fatty acid (MUFA) biosynthesis [8]. SCD1 mainly converts the saturated fatty acids (SFA) palmitic acid (16:0) and stearic acid (18:0) into the MUFAs palmitoleic acid (16:1n7) and oleic acid (18:1n9), respectively [9]. Palmitoleic acid and oleic acid act as signaling molecules, post-translational modifications, sources of energy, and are major components of various lipid classes such as triglycerides, cholesterol esters, and phospholipids [10,11,12,13,14]. Mice globally deficient for *Scd1* (GKO) are protected against obesity in response to HCD, high-fat diet (HFD), and genetically induced adiposity [15,16]. Additionally, mice harboring a liver-specific deletion of *Scd1* (LKO) recapitulate protection from HCD-induced obesity, whereas mice harboring a skin-specific deletion of *Scd1* recapitulate protection from HFD-induced obesity [6,17]. This indicates that the liver is a key organ that regulates whole-body metabolism under high-carbohydrate feeding conditions. Therefore, it is important to understand how hepatic and systemic metabolic pathways are altered by *Scd1* deficiency to produce metabolically favorable phenotypes in both the liver and other organs such as adipose and muscle.

Previously, a microarray analysis of hepatic gene expression was performed on GKO and wild-type mice (WT) fed HCD to determine which genes were differentially expressed. One of the most elevated genes in GKO mice compared to WT mice was insulin-like growth factor-binding protein 1 (*Igfbp1*) [18]. IGFBP1 is a hepatokine that has been reported to enhance β-cell proliferation, insulin signaling, and glucose uptake [19,20,21]. Therefore, IGFBP1 could be contributing to the beneficial effects seen in GKO and LKO mice.

The mechanism leading to IGFBP1 induction in GKO and LKO mice fed HCD is currently unknown, but we hypothesize that fibroblast growth factor 21 (FGF21) signaling is involved. Similar to IGFBP1, FGF21 is a hepatokine that has been shown to increase insulin sensitivity and glucose uptake in muscle and adipose tissue [22,23,24,25]. Moreover, FGF21 signaling can lead to the transcriptional activation of *Igfbp1* [26,27]. In GKO and LKO mice fed HCD, FGF21 expression is significantly elevated, which suggests that FGF21 signaling may be partly responsible for up-regulating IGFBP1. In LKO mice fed HCD, we showed that expression of FGF21 is mediated by peroxisome proliferator-activated receptor-gamma coactivator-1 alpha (PGC-1α) and that PGC-1α expression is dependent upon mTORC1 activation [28,29]. Therefore, we hypothesized that IGFBP1 is regulated by this mTORC1-FGF21 axis.

Global and hepatic *Scd1* deficiency coupled with HCD decreases the ratio MUFAs to SFAs, which leads to the activation of mTORC1 [28]. Specifically, increasing the MUFA levels in GKO mice fed HCD by restoring hepatic oleic acid but not palmitoleic acid biosynthesis suppressed mTORC1 activation [28]. Similarly, supplementing dietary oleic acid, in the form of triolein, to HCD, was shown to suppress FGF21 induction in LKO mice [29]. Lastly, treating LKO primary hepatocytes with oleic acid but not palmitoleic acid suppressed the induction of *Fgf21* [29]. This suggests that if IGFBP1 is regulated by the mTORC1-FGF21 axis then it would also be modulated by the ratio of MUFAs to SFAs.

In this study, we show that IGFBP1 is elevated by global and liver-specific deletion of *Scd1* in mice, as well as by treating a human hepatocyte cell line, with an SCD inhibitor. Moreover, we show that *Igfbp1* induction is blocked by hepatic *Fgf21* deletion, supplementing HCD with triolein, or intraperitoneal injections of mTORC1 inhibitor. These findings were recapitulated in vitro in hepatocytes treated with an SCD inhibitor, mTORC1 inhibitor, shRNA targeting FGF21, and oleic acid. These in vitro treatments blocked *IGFBP1* induction. Taken together, we report that hepatic IGFBP1 is regulated by oleic acid through the mTORC1-FGF21 axis and along with FGF21 could be responsible for enhanced systemic glucose uptake and insulin sensitivity in *Scd1* deficient mice.

## 2. Results

### 2.1. IGFBP1 Expression Is Enhanced by SCD1 Deletion and Inhibition of Enzyme Activity

**Figure 1:**
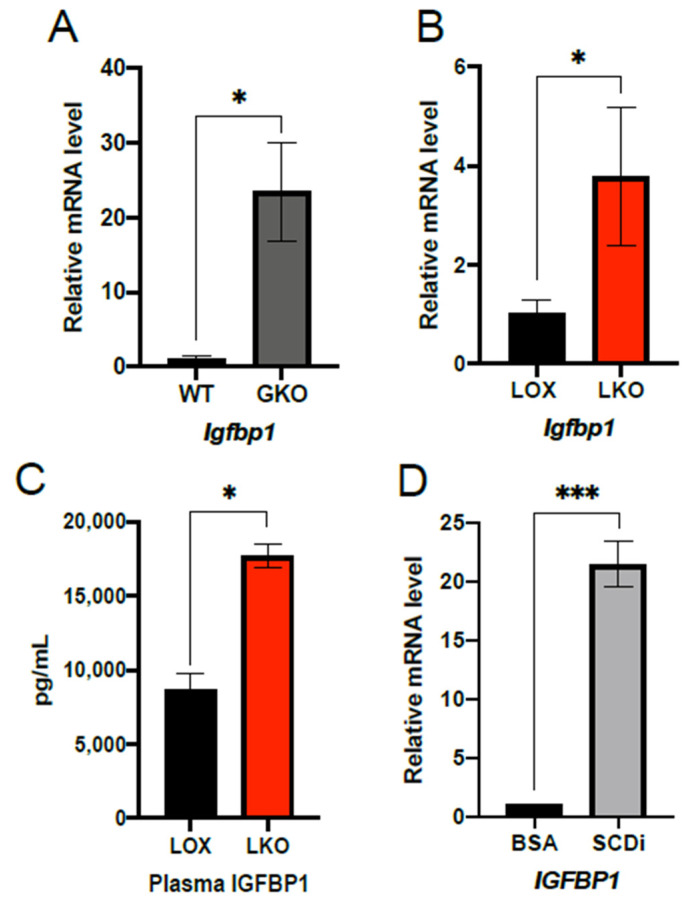
Hepatic SCD1 deficiency up-regulates IGFBP1. (**A**) Relative hepatic *Igfbp1* mRNA levels in 8–10 weeks old male WT (n = 4) and GKO (n = 6) mice fed HCD for 10 days. (**B**) Relative hepatic *Igfbp1* mRNA levels in 8–13 weeks old male LOX (n = 13) and LKO (n = 12) mice fed HCD for 7–10 days. (**C**) Plasma IGFBP1 protein concentration in 8–10 weeks old male LOX (n = 7) and LKO (n = 7) mice fed HCD for 7–10 days. (**D**) Relative *IGFBP1* mRNA levels in HepG2 cells treated for 24 h with BSA (n = 10) or BSA plus 4 μM (n = 12) of SCD inhibitor. Values are mean ± SEM; * *p* < 0.05 vs. WT, LOX, or BSA, *** *p* < 0.005 vs. WT, LOX or BSA by Student’s two-tailed *t*-test.

Previous microarray analysis revealed that *Igfbp1* mRNA was significantly increased in GKO mice fed HCD relative to WT controls [18]. Because GKO mice are protected from HCD-induced obesity, we wanted to assess hepatic *Igfbp1* expression in HCD feeding for 10 days. As expected, *Igfbp1* mRNA transcript levels were significantly elevated in GKO mice compared to WT (Figure 1A). We next assessed if *Igfbp1* was also increased in LKO mice fed HCD. To do this, LKO and LOX control mice were subjected to 7–10 days of HCD, and *Igfbp1* was measured in the liver and plasma. Similar to GKO mice, LKO mice exhibited a significant increase in hepatic *Igfbp1* transcript (Figure 1B). Additionally, LKO mice had significantly elevated plasma protein levels of IGFBP1 (Figure 1C). Lastly, to further support that hepatic deficiency of *Scd1* leads to increased expression of IGFBP1, we assessed *IGFBP1* in HepG2 cells treated with an SCD inhibitor. Consistent with our in vivo results, treating HepG2 cells for 24 h with an SCD inhibitor significantly increased *IGFBP1* mRNA levels (Figure 1D).

### 2.2. Oleic Acid Suppresses IGFBP1 Expression under HCD Feeding Conditions

**Figure 2:**
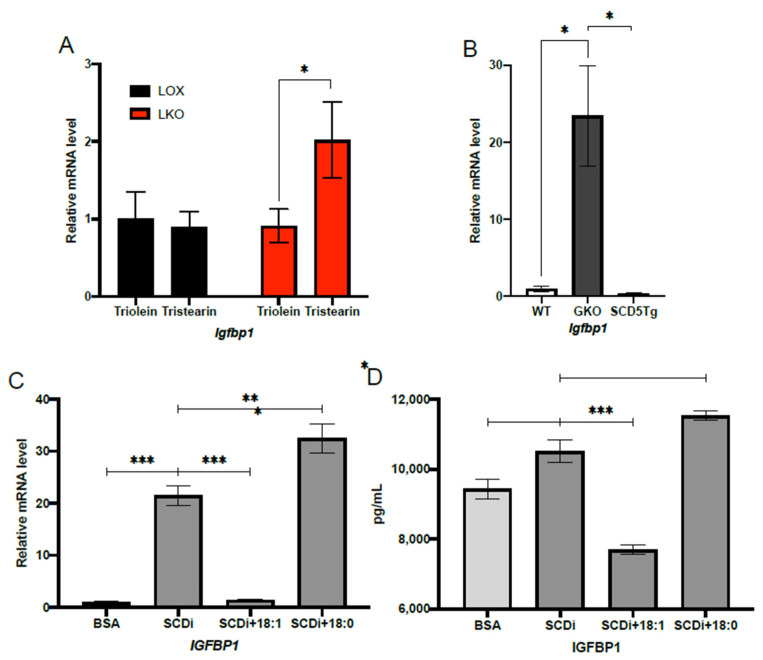
Oleic acid blunts IGFBP1 up-regulation in vivo and in vitro. (**A**) Relative hepatic *Igfbp1* mRNA levels in 12–14 weeks old male LOX and LKO mice fed either HCD supplemented with 15% by weight tristearin (n = 4 LOX and n = 4 LKO) or 15% by weight triolein (n = 5 LOX; 7 LKO) for 10 days. (**B**) Relative hepatic *Igfbp1* mRNA levels in 8–10 weeks old male WT (n = 4), GKO (n = 6), and SCD5tg (n = 5) mice fed HCD for 10 days. (**C**) Relative *Igfbp1* mRNA level in HepG2 cells treated for 24 h with either BSA (n = 10), BSA plus 4 μM SCD inhibitor (n = 12), BSA plus 4μM SCD inhibitor, and 500 μM oleic acid (n = 12), or BSA plus SCD inhibitor and 500 μM steric acid (n = 12). (**D**) Secreted IGFBP1 protein concentration in HepG2 cells treated for 24 h with either BSA (n = 7), BSA plus 4 μM SCD inhibitor (n = 8), BSA plus 4 μM SCD inhibitor and 500 μM oleic acid (n = 7), or BSA plus SCD inhibitor and 500 μM steric acid (n = 8). Values are mean ± SEM; * *p* < 0.05 vs. WT, LOX, or BSA; ** *p* < 0.01 vs. WT, LOX, or BSA; *** *p* < 0.005 vs. WT, LOX triolein or BSA by Student′s two-tailed *t*-test.

To determine if IGFBP1 expression is regulated by the SCD1 product, oleic acid under HCD feeding conditions, we used a combination of dietary, genetic, and cell culture approaches. First, we sought to determine if adding oleic acid in the form of triolein to HCD would be sufficient to blunt the induction of *Igfbp1* in LKO mice. To do this, we fed LKO and LOX mice HCD or HCD supplemented with either triolein or tristearin for 10 days. The LKO mice fed HCD supplemented with triolein exhibited similar *Igfbp1* mRNA levels to LOX mice fed either triolein or tristearin (Figure 2A). In contrast, LKO mice fed HCD supplemented with tristearin exhibited significantly higher mRNA levels of *Igfbp1* compared to LKO mice fed HCD supplemented with triolein or LOX mice fed HCD supplemented with either triolein or tristearin (Figure 2A). This indicates that hepatic oleic acid suppresses the induction of *Igbp1* in LKO mice fed HCD.

Next, we assessed if oleic acid synthesized de novo could also blunt *Igfbp1* induction in LKO mice fed HCD. To restore hepatic de novo synthesis of oleic acid, we used a previously developed transgenic GKO mouse model that expresses human stearoyl-CoA desaturase-5 specifically in the liver (SCD5Tg) [30]. The preferred substrate of hSCD5 is stearic acid, and thus, oleic acid is its predominant product [9]. We subjected WT, GKO, and SCD5Tg mice to 10 days of HCD feeding and measured hepatic *Igfbp1* mRNA levels. Transgenic expression of hSCD5 was able to significantly suppress *Igfbp1* mRNA compared to GKO mice (Figure 2B).

Lastly, to provide further evidence that oleic acid is regulating IGFBP1, we assessed whether oleic acid could suppress the induction of *Igfbp1* in HepG2 cells treated with an SCD inhibitor. Similar to our in vivo results, HepG2 cells treated with an SCD inhibitor and oleic acid, but not steric acid, showed significantly decreased *Igfbp1* mRNA (Figure 2C). In addition, HepG2 cells treated with an SCD inhibitor and oleic acid, but not steric acid, showed significantly decreased secreted IGFBP1 protein levels in the culture media (Figure 2D).

### 2.3. SCD1 Deficiency Enhances IGFBP1 through mTORC1

**Figure 3:**
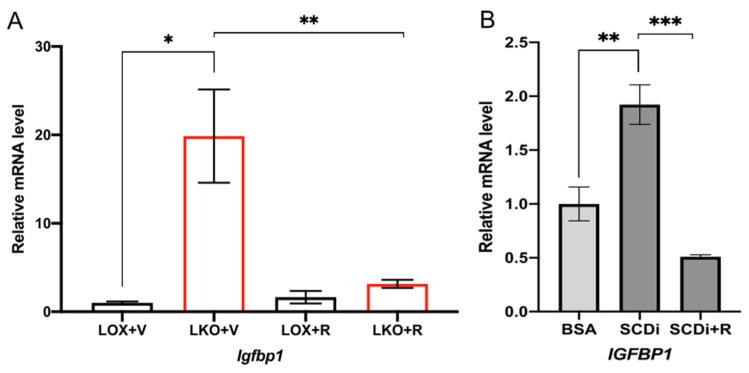
Inhibition of mTORC1 by rapamycin suppresses IGFBP1 induction. (**A**) Relative hepatic *Igfbp1* mRNA levels in 10–12 weeks old male LOX and LKO mice fed HCD diet for 10 days treated daily with either vehicle (n = 3 LOX; 3 LKO) or rapamycin (n = 5 LOX + R; 5 LKO + R). (**B**) Relative *Igfbp1* mRNA levels of HepG2 cells treated with either BSA (n = 5), BSA plus 1μM SCD inhibitor (n = 5), or BSA plus 4 μM SCD inhibitor and rapamycin (n = 5). Values are mean ± SEM; * *p* < 0.05 vs. LOX or BSA; ** *p* < 0.01 vs. LOX+V or BSA; *** *p* < 0.005 vs. LOX or BSA by Student′s two-tailed *t*-test.

We have previously reported that in mice fed HCD, hepatic deletion of *Scd1* leads to activation of the nutrient-sensing kinase mTORC1 and its downstream targets, such as S6 kinase [28]. To determine if IGFBP1 activation by *Scd1* deletion is mTORC1 dependent, we treated LKO mice and SCD1-inhibited HepG2 cells with an mTORC1 inhibitor, rapamycin. In LKO mice fed HCD for 10 days, rapamycin treatment significantly reduced *Igfbp1* expression compared to vehicle-treated controls (Figure 3A). Similarly, in HepG2 cells treated with SCD inhibitor, rapamycin supplementation also significantly reduced *IGFBP1* mRNA levels compared to HepG2 cells treated with SCD inhibitor alone (Figure 3B). This suggests that *Igfbp1* induction by HCD and low levels of oleic acid is mediated by mTORC1.

### 2.4. Oleic Acid Regulates IGFBP1 through the mTORC1-FGF21 Axis

**Figure 4:**
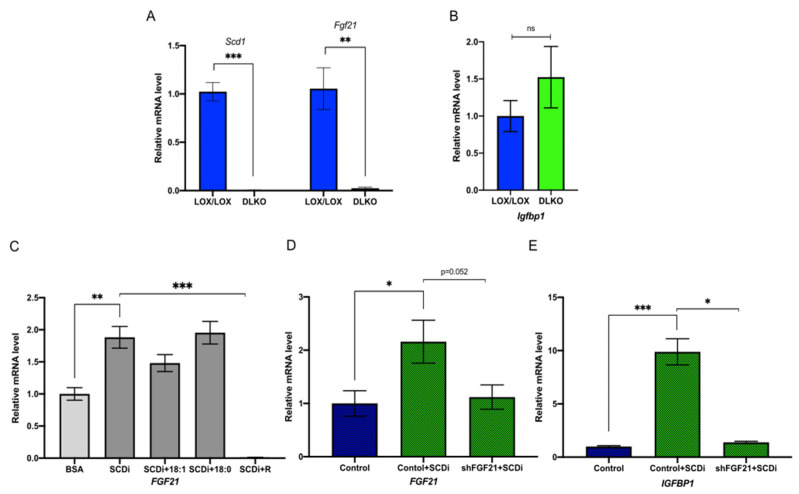
IGFBP1 is regulated by SCD1 through the mTORC1-FGF21 axis. (**A**) Relative hepatic *Scd1* and *Fgf21* mRNA levels in LOX/LOX (n = 6, 3 male; 3 female) and DLKO (n = 6, 3 male; 3 female) mice fed HCD for 10 days. (**B**) Relative hepatic *Igfbp1* mRNA level in LOX/LOX (n = 6, 3 male; 3 female) and DLKO mice fed HCD (n = 6, 3 male; 3 female) for 10 days. (**C**) Relative *Fgf21* mRNA level in HepG2 cells treated with BSA (n = 6), BSA plus 1 μM SCD inhibitor (n = 6), BSA plus 1 μM SCD inhibitor and 100 μM oleic acid (n = 5), BSA plus 1 μM SCD inhibitor and 100 μM steric acid (n = 5), BSA plus 1 μM SCD inhibitor and rapamycin (n = 5). (**D**) Relative *FGF21* mRNA level in HepG2 cells treated with control lentiviral particles (n = 6), control lentiviral particles plus SCD inhibitor (n = 6), or shFGF21 lentiviral particles plus SCD inhibitor (n = 6). (**E**) Relative *IGFBP1* mRNA level in HepG2 cells treated with control lentiviral particles (n = 6), control lentiviral particles plus SCD inhibitor (n = 5), or shFGF21 lentiviral particles plus SCD inhibitor (n = 5). Values are mean ± SEM; ns = not significant; * *p* < 0.05 vs. LOX/LOX, BSA, or control; ** *p* < 0.01 vs. LOX/LOX, BSA, or control; *** *p* < 0.005 vs. LOX/LOX, BSA, or control by Student′s two-tailed *t*-test.

To test whether IGFBP1 is downstream of FGF21 in the mTORC1 axis and if this axis mediates the induction of IGFBP1, we specifically deleted both *Scd1* and *Fgf21* in the livers of mice (DKO) (Figure 4A). In the DKO mice fed HCD for 10 days, there was no significant difference in *Igfbp1* mRNA levels compared to *Scd1*^lox/lox^; *Fgf21*^loxl/lox^ control mice (Figure 4B). To further support these results, we assessed this mechanism in vitro. Using HepG2 cells, we first showed that *Fgf21* is significantly induced by the treatment of SCD inhibitor and that inhibiting mTORC1 with rapamycin blunts *Fgf21* induction (Figure 4C). Similarly, supplementing oleic acid but not steric acid blunts *Fgf21* induction (Figure 4C). Next, we assessed whether the knockdown of *Fgf21* could suppress the induction of *Igfbp1* by SCD inhibition. To do this, HepG2 cells were treated with either lentiviral control particles or shRNA lentiviral particles targeting *Fgf21*, in the presence and absence of an SCD inhibitor. As expected, both *Fgf21* and *Igfbp1* were significantly induced in cells treated with control lentiviral particles and SCD inhibitor compared to cells treated with control lentiviral particles alone (Figure 4D,E). Lastly, treating HepG2 cells with SCD inhibitor and *Fgf21* shRNA lentiviral particles blunted *Fgf21* and *Igfbp1* induction compared to cells treated with control lentiviral particles and SCD inhibitor (Figure 4D,E). Taken together, this suggests oleic acid regulates IGFBP1 through the mTORC1-FGF21 axis.

## 3. Discussion

Global deletion of *Scd1* protects against diet-induced and genetically induced obesity and improves glucose homeostasis [15,16]. Moreover, hepatic deletion of *Scd1* recapitulates protection against HCD-induced obesity [6]. When fed an HCD LKO mice remain lean, insulin-sensitive, and have enhanced glucose uptake in adipose tissue, brown adipose tissue, and liver [29]. The exact mechanism by which SCD1 mediates these beneficial phenotypes remains unclear; however, evidence suggests that hepatokines such as FGF21 and IGFBP1 are contributing factors [20,25,29]. Microarray analysis revealed that *Igfbp1* is one of the most differentially expressed hepatic genes between GKO and WT mice fed HCD, and here we showed that the induction of IGFBP1 is also exhibited in HCD-fed LKO mice. IGFBP1 is a hepatokine that increases β-cell proliferation and enhances insulin signaling [19,20,21]. In humans, a strong positive correlation between circulating IGFBP1 concentrations and insulin sensitivity has been established [31,32,33,34,35,36,37,38]. Likewise, low levels of IGFBP1 have been reported to predict the development of type 2 diabetes and IGFBP1 has been proposed as a potential marker of insulin sensitivity [39,40].

**Figure 5:**
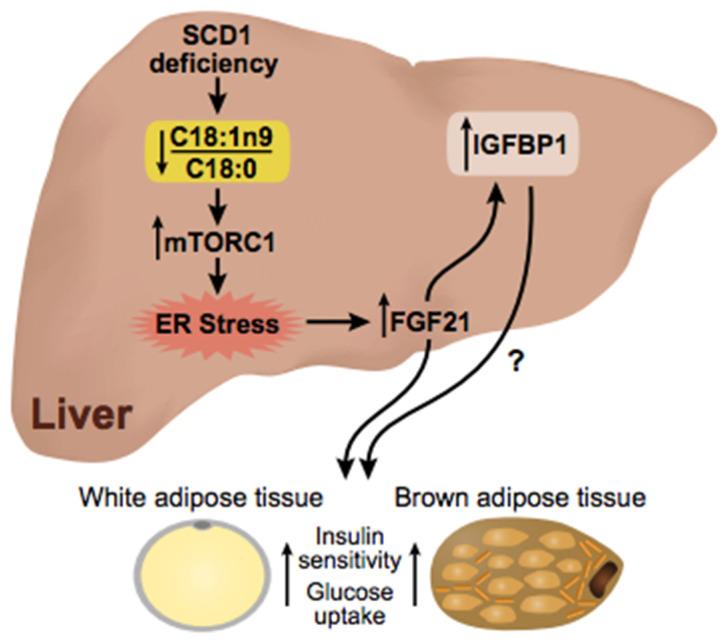
Proposed model of IGFBP1 induction through the mTORC1-FGF21 axis.

In the present study, we sought to determine the mechanism by which *Scd1* deficiency induces *Igfbp1* expression during high-carbohydrate feeding. Previously, our HCD studies revealed that *Scd1* deficiency induces ER stress through SFA activation of the mTORC1-PGC-1α axis [28,41]. Since *Fgf21* and *Igfbp1* are ER stress-inducible genes, their up-regulation is likely in response to ER stress produced by a high ratio of SFA to MUFA levels [42,43]. Increasing the levels of MUFAs relative to SFAs by dietary supplementation or endogenous synthesis of oleic acid inhibits mTORC1 activation and subsequently decreases expression of PGC-1α, FGF21, and ER stress markers [28,29]. Here we provided evidence that IGFBP1 is also regulated by modulating the ratio of MUFAs to SFAs, as well as inhibition of mTORC1 and decreased *Fgf21* expression. Taken together, we show that *Igfbp1* is induced through the mTORC1-FGF21 axis (Figure 5).

Specifically, we showed that *Igfbp1* expression by *Scd1* deficiency is suppressed by dietary supplementation with triolein and endogenously synthesized oleic acid by transgenic expression of hSCD5. Additionally, in cultured HepG2 cells treated with SCD inhibitor, the addition of oleic acid reduces IGFBP1 expression compared to cells treated with SCD inhibitor alone or stearic acid. Moreover, we showed that *Igfbp1* expression is dependent on mTORC1 activation because *Igfbp1* is suppressed by rapamycin treatment of LKO mice or SCD-inhibited HepG2 cells. Lastly, we showed that induction of *Igfbp1* is dependent on FGF21 both in vivo and in vitro. Mice harboring a combined liver-specific deletion of *Scd1* and *Fgf21* fail to significantly induce *Igfbp1* compared to LOX/LOX controls when fed an HCD for 10 days. Similarly, the knockdown of *Fgf21* in SCD-inhibited HepG2 cells blunts the induction of *Igfbp1* compared to cells treated with SCD inhibitor alone.

In conclusion, we report that the hepatokine IGFBP1 is regulated by oleic acid partly through the mTORC1-FGF21 axis. The increased production of IGFBP1 due to hepatic *Scd1* deficiency could be contributing to increased insulin sensitivity and glucose tolerance in *Scd1* deficient mice. More research is needed to determine the effects of IGFBP1 because previous studies have shown conflicting results [44]. If IGFBP1 is acting beneficially, then by reducing the levels of hepatic oleic acid, either by dietary or pharmacological intervention, may be an effective form of treating HCD-induced obesity and the associated metabolic dysfunction.

## 4. Materials and Methods

### 4.1. Animals and Diets

All animal studies were approved by and conducted following the Institutional Animal Care and Use Committee guidelines, University of Wisconsin-Madison, protocol #A005125. Mice were maintained at the University of Wisconsin-Madison animal care facility on regular 12  h light/dark cycles with free access to food and water. All mice used in this study were in the C57BL/6 genetic background and were fed a standard rodent chow diet (Purina 5008; Harlan Teklad, Madison, WI, USA) until being subjected to dietary treatments. All studies were carried out using 8 to 14 weeks old mice. The process of generating *Scd1*^−/−^ (GKO) mice, *Scd1*^−/−^; *SCD5*Tg + (SCD5Tg), *Scd1*^lox/lox^ (LOX), *Scd1*^lox/lox^; Albumin Cre/+, has been described previously [6,15,30]. To generate *Scd1* and *Fgf21* liver-specific double knockout mice, *Scd1*^lox/lox^; Albumin Cre/+ were crossed to *Fgf21*^lox/lox^ mice obtained from The Jackson Laboratory (Fgf21^tm1.2Djm^, JAX stock #022361) to yield *Scd1*^lox/+^; *Fgf21*^lox/+^*;* Albumin Cre/+ and *Scd1*^lox/+^; *Fgf21*^lox/+^. These mice were subsequently crossed to yield *Scd1*^lox/lox^; *Fgf21*^loxl/lox^*;* Albumin Cre/+. For dietary studies, mice were fed a very-low-fat high-carbohydrate diet (HCD; Harlan Teklad, Madison, WI, USA TD.03045) for a period of 7–14 days. Triolein or tristearin-supplemented HCDs were prepared by supplementing the fat-free basal mix (TD150776.PWD; Harlan Teklad, Madison, WI, USA) with 15% by weight of tristearin (T5016; Sigma, St. Louis, MO, USA) or triolein (99% purity, T7140; Sigma). For rapamycin studies, mice were treated intraperitoneally with 2  mg/kg/day of rapamycin (from LC laboratories, Woburn, MA, USA) dissolved in saline and 2% ethanol for 10 days. All mice were fasted for 4  h before being euthanized by isoflurane overdose. Collected tissues and plasma were frozen in liquid nitrogen and stored at −80 °C for future analysis. Plasma IGFBP1 protein levels were measured using the Mouse IGFBP-1 ELISA Kit PicoKine™ (Boster Biological Technology, Pleasanton, CA, USA, Catalog #EK0383).

### 4.2. Quantitative Real-Time PCR

Total RNA was isolated using the RNeasy Lipid Tissue Mini Kit (Qiagen) for animal tissues and the Direct-zol RNA microprep kit (Zymo Research, Irvine, CA, USA) for cell culture samples. cDNA was synthesized from RNA using the ABI High Capacity cDNA Reverse Transcription Kit (Life Technologies/Invitrogen, Carlsbad, CA, USA). qPCR was performed on an ABI7500 instrument using the ABI Fast SYBR Green Master Mix (Life Technologies/Invitrogen, Carlsbad, CA, USA) for animal tissues and 2x SYBR Green qPCR Master Mix (Bimake, Houston, TX, USA) for cell culture samples. Relative mRNA abundance was calculated as relative *C_t_* value and normalized to 18 s for animal tissues and GAPDH for cell culture samples by the ΔΔ*C_t_* method. Primer sequences are available upon request.

### 4.3. Cell Culture and Treatments

HepG2 cells were cultured in Dulbeccos’s Modified Eagle Medium (DMEM) supplemented with 10% fetal bovine serum (FBS), penicillin (100 U/mL), and streptomycin (100 μg/mL). Upon confluence, the HepG2 cells were serum-starved for 24 h before various treatments. To model SCD1 deficiency, HepG2 cells were treated with 1 or 4 μM of SCD inhibitor A939572 (Cayman Chemical, MI, USA) for 24 h. To inhibit mTORC1, HepG2 cells were treated with 100 nM of rapamycin (APExBIO, Houston, TX, USA) for 24 h.

Oleic acid and stearic acid were conjugated to fatty acid free bovine serum albumin to create 100 or 500 mM stock solutions, respectively. These stock solutions were subsequently diluted to 100 or 500 μM with DMEM without FBS, penicillin, and streptomycin, and added directly to the HepG2 cells for 24 h. Fatty acid treatment between 50–500 μM is within the physiologically relevant range of human and mouse models [45,46]. A 0.17 mM solution of fatty acid free BSA and PBS was prepared by first warming PBS to 37 °C on a heated stir plate. Next, fatty acid free BSA was slowly added to PBS under gentle stirring until it was completely dissolved. To create the 100 and 500 mM stock solutions, fatty acid was weighed out and dissolved in hexane. Next, the dissolved fatty acid was transferred into a glass vial and dried under a stream of nitrogen gas. The fatty acid free BSA solution was added to the vial and gently stirred for 4 h at 37 °C. This was created either 1 mM fatty acid to 0.17 mM BSA solution with a 6:1 molar ratio of fatty acid to BSA or 5 mM fatty acid to 0.017 mM BSA with a 30:1 molar ratio of fatty acid to BSA.

Treatment of HepG2 cells with FGF-21 shRNA lentiviral particles (Santa Cruz Biotechnology, Dallas, TX, USA; sc-39484-V) and copGFP control lentiviral particles (Santa Cruz Biotechnology, Dallas, TX, USA; sc-108084) was performed according to the manufacturer’s instructions. Briefly, cells were grown to 50% confluence in 6-well plates and treated with 20 μL of lentiviral particles and 2 μg/mL polybrene for 24 h. After 24 h, the media was removed and replaced with complete media with and without SCD inhibitor. Lastly, secreted IGFBP1 protein was measured using the Human IGFBP-1 ELISA Kit PicoKine™ (Boster Biological Technology, Pleasanton, CA, USA, Catalog #EK0382).
